# Impact of the metabolic syndrome on prevalence and survival in motor neuron disease: a retrospective case series

**DOI:** 10.1007/s11011-023-01296-2

**Published:** 2023-09-25

**Authors:** Jee Eun Oh, Jee Ah Oh, Mary Demopoulos, Karen M Clark, Matthew CL Phillips

**Affiliations:** Waikato Hospital, Hamilton, New Zealand

**Keywords:** Motor neuron disease, Amyotrophic lateral sclerosis, Metabolic syndrome, Prevalence, Survival, Ethnicity

## Abstract

Metabolic dysfunction is an important factor in the pathogenesis of motor neuron disease, but its prevalence and association with survival in this disorder is unknown. We hypothesized that patients with motor neuron disease would show a higher prevalence of metabolic syndrome compared to the general New Zealand population, and that metabolic syndrome would be associated with worsened survival. We undertook a retrospective analysis in 109 motor neuron disease patients diagnosed and treated at Waikato Hospital from 2013 to 2020. Demographic, clinical, and laboratory data were collected. Survival was defined as the date of initial symptom onset to the date of death. Of 104 eligible patients, 34 patients (33%) had metabolic syndrome (33% of Europeans, 46% of Māori). Mean survival in motor neuron disease patients with metabolic syndrome was significantly reduced compared to patients without metabolic syndrome (38 vs. 61 months, *P* = 0.044), with a 5-year survival rate of 21% for the former and 38% for the latter (*P* = 0.012). Compared with the general New Zealand population, metabolic syndrome is highly prevalent amongst motor neuron disease patients in the Waikato region and it is associated with worsened survival. Metabolic dysfunction may be a key factor underlying the pathogenesis of motor neuron disease.

## Introduction

Motor neuron disease (MND) encompasses a collection of clinical disorders that are characterized by progressive muscle weakness and motor neuron degeneration on electrophysiological testing (Masrori and Damme 2020). The most common form of MND is amyotrophic lateral sclerosis (ALS), with other forms including primary lateral sclerosis, progressive muscular atrophy, pseudobulbar palsy, and progressive bulbar palsy. ALS carries a particularly poor prognosis, with most patients dying between 24 and 50 months after the onset of symptoms (Longinetti and Fang 2019).

Impaired mitochondrial metabolism is increasingly recognized as a key factor in the pathogenesis and progression of ALS (Blasco et al. 2020; Dupuis et al. 2011; Kiernan et al. 2011; Kodavati et al. 2020; Obrador et al. 2021; Singh et al. 2021; Smith et al. 2019; Tefera and Borges 2016). Mitochondria dysfunction is associated with inefficient energy production, excess oxidative stress, neuroinflammation, excitotoxicity, axonal transport defects, and the activation of apoptotic pathways (Manfredi and Xu 2005; Xiao et al. 2018). Given these metabolic abnormalities, patients who develop MND might be anticipated to show a higher prevalence of the metabolic syndrome, which has a prevalence of 16% in the general New Zealand (NZ) European population, with a particularly high prevalence of 32% in the Māori population (Gentles et al. 2007). Moreover, given that patients with metabolic syndrome already show evidence of metabolic and mitochondria dysfunction (Prasun 2020), MND progression in these patients might be expected to be more rapid, leading to worsened survival. To our knowledge, the association between metabolic syndrome and MND survival has not been investigated.

On this background, we conducted a retrospective case series analysis amongst MND patients in a NZ cohort to determine whether patients with MND showed a higher prevalence of metabolic syndrome compared to the general NZ population, and whether metabolic syndrome was associated with worsened survival in MND. We also looked at ethnic differences between outcomes.

## Methods

We conducted a retrospective analysis of all MND patients registered in the Neurology Department at Waikato Hospital from June 2013 to June 2020. Waikato Hospital serves a population of nearly half a million people, which includes a higher proportion of Māori compared to the national average. All procedures performed in this study were prospectively approved by the Central Health and Disability Ethics Committee of NZ.

A variety of demographic, clinical, and biochemical data were collected using patient clinical records. All patients were diagnosed with MND by a neurologist, which included identification of the subtype as either ALS, primary lateral sclerosis, progressive muscular atrophy, pseudobulbar palsy, or bulbar palsy. Socio-economic status was determined from the patient’s address using one of the three NZ Index of Deprivation scores (2006, 2013, or 2018, whichever one was closest to the symptom onset for each patient) (EHINZ 2023). Occupation was divided into 11 groups using the Australia and NZ Standard Classification of Occupation 2013, Version 1.2 (Australian Bureau of Statistics 2023).

Metabolic syndrome was defined in accordance with the Adult Treatment Panel (NCEP-ATPIII) criteria as the presence of three or more of the following - high body mass index (BMI) (defined as 30 kg/m^2^ or higher), glucose intolerance (defined either on medical history and/or by a glycated haemoglobin (HbA1c) over 40 mmol/mol), hypertension, elevated triglycerides (defined as 1.7 mmol/L or higher), elevated high-density lipoprotein (HDL) (defined as less than 1 mmol/L), and the use of statins. We did not use waist circumference, as this data was not readily available for our patients. Central obesity was assumed if the BMI was above 30 kg/m^2^, as supported by the International Diabetes Federation (Alberti et al. 2006). Glucose intolerance is defined as fasting glucose of 6.1mmol/L or higher in NCEP-ATPIII, but since this data was not commonly available among our patients, the HbA1c was used as a surrogate marker (which may also be a better reflection of longer-term glucose intolerance). We used an HDL cut-off of less than 1 mmol/L for everyone (instead of 1.04 mmol/L for men and 1.29 mmol/L for women) to simplify data collection.

Comparisons for a variety of risk factors between patients with and without metabolic syndrome, as well as between NZ Europeans and Māori, were calculated using student’s t-tests and chi-squared tests as appropriate. Kaplan-Meier estimator and Cox proportional hazards models were used to analyze survival, which was defined as the interval between symptom onset and death. Statistical significance was defined at a *P*-value of ≤ 0.05.

## Results

Baseline demographic, metabolic, clinical, management, and survival data are presented in Table 1. 109 patients were identified from the departmental database, of which 5 patients were excluded (2 patients did not actually have a diagnosis of MND, 1 patient could not be accessed due to an incorrect national health identifier number, and 2 patients lacked metabolic data). Of 104 eligible patients, 34 patients (33%) had metabolic syndrome (33% of NZ Europeans, 46% of Māori). Patients with metabolic syndrome had a mean survival of 38 months, which was significantly reduced compared to 61 months for patients without metabolic syndrome (*P* = 0.044). When assessed individually, the components of the metabolic syndrome were not associated with any significant difference in survival. There were 85 patients (82%) of European ethnicity and 11 patients (11%) of Māori ethnicity. European patients with MND had a mean survival of 50 months, which was significantly lower compared to 94 months for Māori patients (*P* = 0.013).

**Table 1:** Baseline demographic, metabolic, clinical, management, and survival data

	With metabolic syndrome (n = 34)	Without metabolic syndrome (n = 70)	*P*	European (n = 85)	Māori (n = 11)	*P*	Total (n = 104)
**Demographic**							
Gender							
Male	21	43	0.974	51	8	0.414	64
Female	13	27		34	3		40
Ethnicity							
European	28	57	0.411	-	-	-	85
Māori	5	6		-	-		11
Other	1	7		-	-		8
Age (years, mean)							
At onset	68.9	62.4	0.007	66.7	51.5	< 0.001	64.6
At diagnosis	70.4	64.2	0.007	68	56.2	< 0.001	66.3
Residence							
Large urban	11	29	0.642	32	5	0.497	40
Small urban	19	35		34	4		54
Rural	4	6		8	2		10
Socio-economic status							
NZ Dep 1–3	9	19	0.919	27	0	0.002	28
NZ Dep 4–7	12	27		33	2		39
NZ Dep 8–10	13	24		25	9		37
Occupation							
Non-farming managers	5	9	0.285	12	1	0.094	14
Farmers	2	3		5	0		5
Professionals	1	7		7	0		8
Technicians and trades	2	13		12	1		15
Community and postal	3	3		6	0		6
Administrative	2	7		9	0		9
Sales	1	2		2	1		3
Machinery and drivers	2	3		4	1		5
Labourers	2	5		5	1		7
Non-labour force	3	0		1	2		3
Unidentifiable	11	18		22	4		29
**Metabolic**							
Metabolic syndrome							
BMI > 30 at baseline	16	13	0.005	22	5	0.05	29
Glucose intolerance	14	8	< 0.001	20	2	0.701	22
Hyperlipidemia or statin	31	55	0.124	69	11	0.312	86
Hypertension	28	13	< 0.001	34	3	0.398	41
Metabolic syndrome	-	-	-	28	5	0.411	34
BMI (kg/m^2^, mean +/- SD)							
At onset	30.7 +/- 5.1	27.1 +/- 4.4	< 0.001	28 +/- 4.8	32.4 +/- 5.4	0.019	28.3 +/- 4.9
At diagnosis	28.4 +/- 5.2	25.3 +/- 4.1	0.003	26 +/- 4.4	30.5 +/- 6.3	0.010	26.4 +/- 4.7
Other factors							
Stroke	6	0	< 0.001	4	1	0.545	6
Ischemic heart disease	9	2	< 0.001	10	1	0.784	11
Smoking	16	33	0.881	38	7	0.448	49
Alcohol	17	33	0.798	38	8	0.094	50
**Clinical**							
Sporadic or familial							
Sporadic	33	65	0.568	81	10	0.311	98
Familial	1	2		3	0		3
Unknown	0	3		1	1		3
Subtype							
Limb-onset ALS	15	36	0.198	43	4	0.131	51
Bulbar-onset ALS	16	19		29	3		35
PLS	2	6		6	2		8
PMA	0	5		2	2		5
PBP	1	1		2	0		2
Unknown	1	3		3	0		3
FTD							
FTD diagnosis	0	3	0.472	3	0	0.404	3
**Management**							
Investigations							
Electromyography	23	50	0.692	59	9	0.394	73
MRI cervical spine	25	40	0.105	50	10	0.123	65
Treatments							
Riluzole	12	23	0.805	30	2	0.257	35
Non-invasive ventilation	7	18	0.540	18	5	0.080	25
Gastrostomy	18	34	0.726	45	3	0.101	52
**Survival**							
Duration (months, mean +/- SD)							
Onset to death	38 +/- 37	61 +/- 58	0.044	50 +/- 47	94 +/- 89	0.013	54 +/- 53

*NZ Dep = New Zealand deprivation score; BMI = body-mass index; ALS = amyotrophic lateral sclerosis; PLS = primary lateral sclerosis; PMA = progressive muscular atrophy; PBP = progressive bulbar palsy; FTD = frontotemporal dementia; MRI = magnetic resonance imaging

*Given that data for patients of other ethnicities are not shown, the values for European and Māori may not add up to the total in the far-right column

**Fig. 1:**
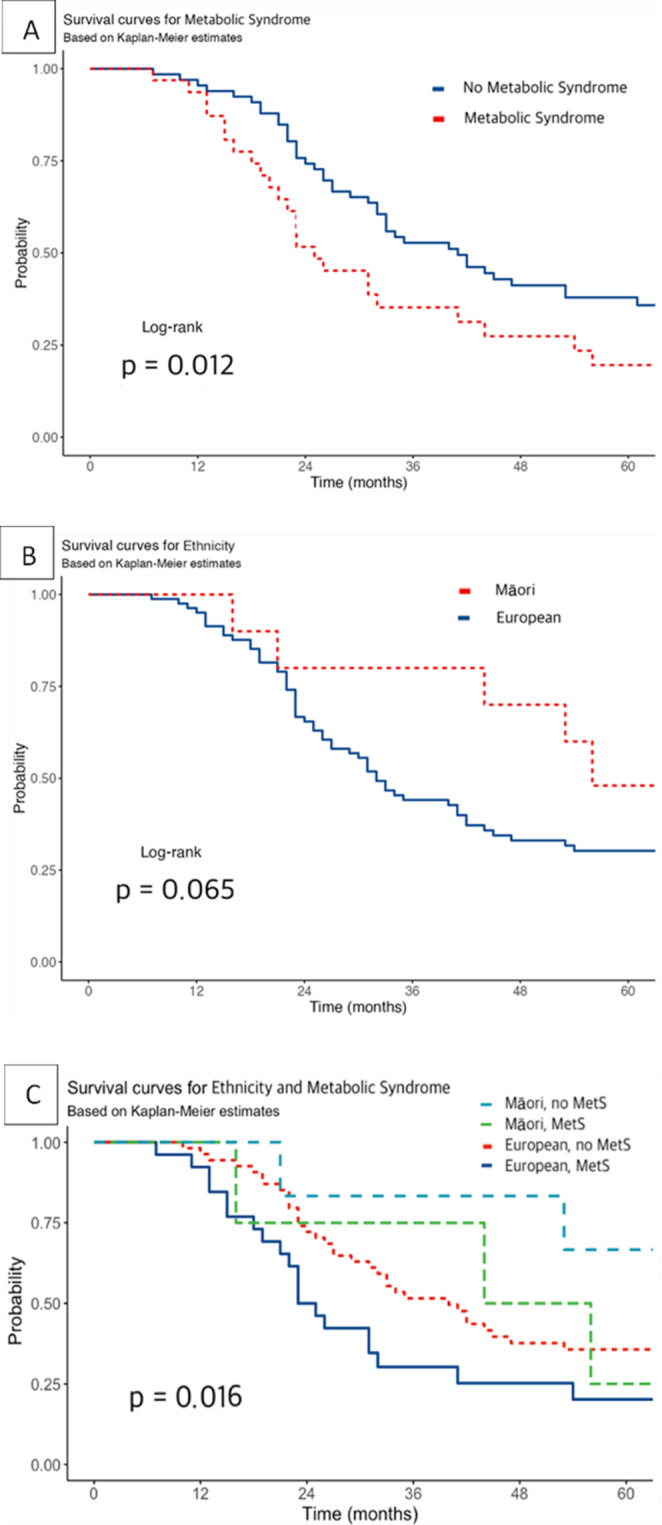
Kaplan-Meier survival curves, showing (**A**) survival in MND patients with metabolic syndrome compared to MND patients without metabolic syndrome, (**B**) survival in NZ European MND patients compared to Māori patients, and (**C**) survival in MND patients with and without metabolic syndrome with respect to ethnic breakdown

Adjusted 5-year-survival rates are shown in Fig. 1. Figure 1 A shows significantly worsened survival in MND patients with metabolic syndrome compared to MND patients without metabolic syndrome, with the 5-year survival rate for patients with metabolic syndrome only 21%, compared to 38% for patients without metabolic syndrome (*P* = 0.012). The unadjusted hazard ratio for patients with metabolic syndrome was 1.81 (1.13–2.88, *P* = 0.013), but when adjusted for age, gender, and ethnicity, the hazard ratio was 1.68 (1.02–2.77, *P* = 0.041). Figure 1B shows a non-significant trend towards improved survival in NZ Māori patients compared to European patients (*P* = 0.065). Figure 1 C shows that the survival difference between patients with and without metabolic syndrome persisted with the ethnic breakdown, with NZ European patients with metabolic syndrome showing the worst survival (*P* = 0.016). All patients died from terminal complications related to their MND, except for 1 patient who died from a myocardial infarction.

## Discussion

In this retrospective case series analysis, we found the prevalence of metabolic syndrome to be higher in our NZ cohort of MND patients compared with the general NZ population. Moreover, the presence of metabolic syndrome was associated with significantly worsened survival in MND, even after adjustment for age, gender, and ethnicity.

In our MND cohort, 34 patients (33%) had metabolic syndrome (33% of NZ Europeans, 46% of Māori), which is relatively high compared with the general NZ prevalence of 26% (16% of Europeans, 32% of Māori) that was observed by a large cross-sectional survey (Gentles et al. 2007). The much higher prevalence of metabolic syndrome amongst NZ Europeans in our MND cohort, compared to a neighbouring population, was striking. Although speculative, this finding may indicate that patients with metabolic syndrome are somehow metabolically predisposed to developing MND. Given the disposition of both metabolic syndrome and MND towards prominent metabolic and mitochondrial abnormalities, mitochondria dysfunction may constitute the underlying, shared pathology that predisposes a person to both disorders.

In our MND cohort, mean survival was significantly reduced in patients with metabolic syndrome compared to patients without metabolic syndrome (38 vs. 61 months). Even after adjusting for age, gender, and ethnicity, 5-year survival rates were significantly worse amongst the metabolic syndrome patients compared to those without metabolic syndrome (21% vs. 38%, hazard ratio 1.68). This considerable difference in survival time suggests a patient’s metabolic status may influence the rate of their MND progression. It has long been known that metabolic and mitochondria dysfunction are present during the early stages of ALS, which is associated with glucose intolerance (Ionǎşescu and Luca 1964; Nagano et al. 1979; Reyes et al. 1984), as well as inefficient adenosine triphosphate (ATP) production, increased oxidative stress, and a reduced ability to maintain energy and protein homeostasis (Smith et al. 2019; Valbuena et al. 2016), all of which could contribute to a more rapid progression of the degenerative process. Despite the substantial evidence of metabolic dysfunction in ALS, there have been conflicting reports on the effect of metabolic risk factors on ALS incidence and prognosis (Brito et al. 2019; Chiò et al. 2009; Dupuis et al. 2008; Kioumourtzoglou et al. 2015; O’Reilly et al. 2013; Mitchell et al. 2015; Moglia et al. 2017; Rafiq et al. 2015; Seelan et al. 2014; Sun et al. 2015; Tsai et al. 2019). However, most of these studies assessed individual factors without considering their combined interactive effect, and many of them performed little or no adjustment for factors known to affect incidence and survival (such as age, gender, and ethnicity). When assessed individually, the components of metabolic syndrome in our cohort were not associated with a significant difference in survival, which suggests that an overall combined metabolic risk profile, rather than individual risk factors, is the crucial factor associated with a worsened outcome. Importantly, nearly all the patients in our cohort passed away due to progression of their MND, rather than an unrelated disorder (except 1 patient, who died of a myocardial infarction rather than their MND), which argues against the possibility that patients with metabolic syndrome died as a result of the metabolic syndrome itself.

Although we detected a significant mean survival difference between NZ European and Māori patients with MND (50 vs. 94 months), the 5-year survival rates showed a non-significant (albeit borderline) trend towards prolonged survival in the latter population. This trend was an unexpected finding, particularly since the Māori patients in our cohort showed a higher prevalence of metabolic syndrome compared with European patients. Although this finding is interesting, given the small number of Māori patients in our cohort (11 patients), it is difficult to draw definitive conclusions about any potential impact of ethnicity on survival.

There were several strengths to this study. It is the first study to examine metabolic syndrome and its association with MND. Moreover, despite using stringent criteria for defining metabolic syndrome in our study population, we nevertheless found a significantly increased prevalence of metabolic syndrome in our cohort compared to the general NZ population.

Our study also had several limitations. First, since retrospective studies are unable to identify causal relationships, we are unable to comment on causality. Second, there were some limitations with respect to how the metabolic data was collected - given that it is not standard practice in our neurology department for MND patients to have either their weight circumference or fasting glucose level measured, we had to use surrogate markers, such as BMI (as a substitute for waist circumference) and HbA1c (for the fasting glucose level). Third, given the low number of Māori patients in our cohort, there was insufficient power to make any firm conclusions with respect to the observed survival differences based on ethnicity.

In conclusion, we have shown that metabolic syndrome is highly prevalent amongst MND patients in the Waikato region of NZ compared with the general NZ population. Moreover, metabolic syndrome is associated with considerably worsened survival in MND. These findings strengthen the possibility that metabolic and mitochondria dysfunction may be the crucial factors underlying the pathogenesis of MND. Although further observational studies are needed to corroborate our findings, prospective clinical trials will be crucial to determine whether therapies aimed at enhancing mitochondria function are capable of mitigating the pathological process in MND.

## Data Availability

The datasets generated during and/or analyzed during the current study are available from the corresponding author on reasonable request.
